# Neural basis of working memory in ADHD: Load versus complexity

**DOI:** 10.1016/j.nicl.2021.102662

**Published:** 2021-04-03

**Authors:** Prerona Mukherjee, Tadeus Hartanto, Ana-Maria Iosif, J. Faye Dixon, Stephen P. Hinshaw, Murat Pakyurek, Wouter van den Bos, Amanda E. Guyer, Samuel M. McClure, Julie B. Schweitzer, Catherine Fassbender

**Affiliations:** a Department of Psychiatry and Behavioral Sciences and MIND Institute, University of California, Davis, 2825 50th St., Sacramento, CA 95817, USA; b Department of Public Health Sciences, University of California, Davis, Davis, CA 95616, USA; c Department of Psychology, University of California, Berkeley, 3rd Floor, Berkeley Way West Building, 2121 Berkeley Way West, Berkeley, CA 94720, USA; d Department of Developmental Psychology, University of Amsterdam, Nieuwe Achtergracht 129-B, 1018 WS Amsterdam, Netherlands; e Department of Human Ecology, University of California, Davis, 1 Shields Ave, Davis, CA 95616, USA; f Center for Mind and Brain, University of California, Davis, 267 Cousteau Pl, Davis, CA 95618, USA; g Department of Psychology, Arizona State University, Tempe, AZ 85287, USA; h School of Psychology, Dublin City University, DCU Glasnevin Campus, Dublin 9, Ireland

**Keywords:** Functional imaging, fMRI, Working memory capacity, ADHD, Caudate, Cerebellum, Prefrontal cortex

## Abstract

Working memory (WM) deficits are key in attention deficit hyperactivity disorder (ADHD). Nevertheless, WM is not universally impaired in ADHD. Additionally, the neural basis for WM deficits in ADHD has not been conclusively established, with regions including the prefrontal cortex, cerebellum, and caudate being implicated. These contradictions may be related to conceptualizations of WM capacity, such as load (amount of information) versus operational-complexity (maintenance-recall or manipulation). For instance, relative to neurotypical (NT) individuals, complex WM operations could be impaired in ADHD, while simpler operations are spared. Alternatively, all operations may be impaired at higher loads. Here, we compared the impact of these two components of WM capacity: load and operational-complexity, between ADHD and NT, behaviorally and neurally. We hypothesized that the impact of WM load would be greater in ADHD, and the neural activation would be altered. Participants (age-range 12–23 years; 50 ADHD (18 females); 82 NT (41 females)) recalled three or four objects (load) in forward or backward order (operational-complexity) during functional magnetic resonance imaging scanning. The effects of diagnosis and task were compared on performance and neural engagement. Behaviorally, we found significant interactions between diagnosis and load, and between diagnosis, load, and complexity. Neurally, we found an interaction between diagnosis and load in the right striatum, and between diagnosis and complexity in the right cerebellum and left occipital gyrus. The ADHD group displayed hypo-activation compared to NT group during higher load and greater complexity. This informs mechanisms of functional problems related to WM in adolescents and young adults with ADHD (e.g., academic performance) and remedial interventions (e.g., WM-training).

## Highlights

• Working memory (WM).
• Load versus Complexity.
• ADHD.
• FMRI.
• Working Memory Striatum and Cerebellum.

## 1 Introduction

Attention Deficit Hyperactivity Disorder (ADHD) is a common, early-onset neurodevelopmental disorder, with an estimated prevalence of 5–6%, often persisting into adulthood (Asherson et al., 2016). A prominent deficit in ADHD is working memory (WM), with some research suggesting that WM may be a core impairment in ADHD (Martinussen et al., 2005, Rapport et al., 2001). WM impairments are linked to key symptoms such as inattention and hyperactivity in ADHD (Orban et al., 2018, Rapport et al., 2009, Campez et al., 2020). WM capacity refers to the ability to maintain or manipulate information mentally, following perceptual input (Baddeley et al., 1974). Undeniably, WM-related impairments can have a profound influence on a variety of functions, affecting areas of life, such as academic achievement (Simone et al., 2018, Fried et al., 2019), emotion processing (Groves et al., 2020), social relationships (Kofler et al., 2011). Therefore, a more comprehensive understanding of WM-related impairment in ADHD could have important implications.

One of the complicating factors in WM research is differences in defining WM constructs. Some WM theories differentiate between maintenance and manipulation, qualifying only manipulation as true WM, with maintenance being simply recall (Rapport et al., 2013), whereas others consider both to be WM operations of varying complexity (D'Esposito et al., 1999, Rypma et al., 2002, Jolles et al., 2011). The fidelity of information stored in WM is reduced as the complexity of the operations being performed on the information increases (e.g., maintenance versus manipulation). A similar negative effect on WM is observed as the amount of information maintained (i.e., load) increases. Thus, WM capacity may be affected by load, operational-complexity, or both.

Divergent models have been proposed to explain the neural basis of different WM constructs. One model of WM posits that maintenance and manipulation rely on different networks in frontal and parietal cortex. Maintenance is thought to recruit a more ventral network, whereas manipulation relies additionally on more dorsal regions (D'Esposito et al., 1999, Crone et al., 2006). However, at higher loads, maintenance has also been demonstrated to engage dorsal networks (Rypma et al., 2002, Miller, 1956, Braver et al., 1997, Tan et al., 2006, Jaeggi et al., 2009, Zarahn et al., 2005). Thus, manipulation could be perceived as a high-load WM task rather than a dissociable component with a dedicated brain network*.* Very few studies have tested this by directly comparing maintenance at higher load with manipulation (Jolles et al., 2011, Veltman et al., 2003, Cannon et al., 2005). Two such studies found maintenance at higher load recruited similar regions as manipulation, including the dorsolateral prefrontal cortex (DLPFC) (Veltman et al., 2003, Cannon et al., 2005) while another found no DLPFC recruitment for manipulation (Jolles et al., 2011). Other work shows WM capacity, especially the ability to perform manipulation, is supported by corresponding DLPFC activation and increases with age (Jolles et al., 2011, Crone et al., 2006, Federico et al., 2014).

WM deficits are key in ADHD (Martinussen et al., 2005, Rapport et al., 2001). WM is linked to ADHD symptoms (Rapport et al., 2009), and WM deficits persist into adulthood (Alderson et al., 2013). Nevertheless, WM is not universally impaired in ADHD (Martinussen et al., 2005, Rapport et al., 2008, Gathercole and Alloway, 2006, Vance et al., 2013, Kofler et al., 2019, Nigg, 2005), and this heterogeneity is not fully understood. Other complicating factors may include the possibility that WM impairments in ADHD may be modality-specific. It is possible that spatial WM might be more affected than verbal (Martinussen et al., 2005). However, a recent meta-analysis found verbal WM to be impacted in ADHD (Ramos et al., 2020). Other theories suggest that WM may be impacted more in individuals with inattentive symptoms (Martinussen and Tannock, 2006), yet WM deficits are also associated with hyperactive/impulsive symptoms (Kofler et al., 2019).

In the current study, we propose that ADHD-related alterations of WM could depend on whether WM capacity is defined by load and/or complexity. Thus, complex WM operations, such as manipulation, could be impacted in ADHD, with simpler operations, such as maintenance-recall, might be less affected, as in conditions like Parkinson’s disease (Lewis et al., 2003). Alternatively, both manipulation and maintenance at higher loads could be impaired, as is observed in schizophrenia (Cannon et al., 2005, Hill et al., 2010).

The neural basis for WM deficits in ADHD could further contribute to heterogeneity in findings regarding WM impairments in ADHD. The brain networks supporting WM in neurotypical (NT) individuals have been studied extensively, and while the prefrontal cortex (PFC), parietal cortex (PC), supplementary motor area (SMA) and superior temporal areas (D'Esposito et al., 1999) are classically linked to WM, recent studies suggest the cerebellar (Tomlinson et al., 2014, Steinlin, 2007) and striatal regions (O'Reilly and Frank, 2006, Darki and Klingberg, 2015) play essential roles in WM processing. The striatum is linked to gating information in the PFC (Chatham and Badre, 2015, McNab and Klingberg, 2008), and thus is critical to WM capacity (e.g., maintenance) while the cerebellum is engaged with increased complexity (Marvel and Desmond, 2012) (e.g., manipulation). Structural differences have been reported in both the caudate (Vaidya, 2012, Valera et al., 2007, Hoogman et al., 2017) and cerebellum (Steinlin, 2007, Vaidya, 2012, Valera et al., 2007, Baldaçara et al., 2008, Berquin et al., 1998, Giedd et al., 2001, Casey et al., 2007) in ADHD, as compared to NT, and key reviews of WM impairments in ADHD have suggested fronto-striato-cerebellar networks could play a key role in WM deficits in ADHD (Martinussen et al., 2005, Giedd et al., 2001, Castellanos et al., 2002, Durston, 2003, Bollmann et al., 2017). Thus, WM impairments in ADHD could be driven by either increases in load or complexity, via differences in recruitment of striatal or cerebellar systems in connection with frontal networks. Therefore, in addition to investigating differences in WM performance, examination of the neural basis for WM impairments in ADHD, whether driven by load or complexity, would enable identification of the locus for WM differences in ADHD.

To directly compare the impact of different definitions of WM capacity in ADHD, we tested the effect of WM load (low versus high) and complexity (maintenance-recall versus manipulation) within a unitary fMRI paradigm, in a group of individuals with ADHD and a NT control group. We hypothesized that WM performance would be impaired in the ADHD compared with the NT group and that this difference in performance would be accompanied by alterations in WM-related neural activation. Furthermore, based on the results of previous behavioral studies examining the impact of WM load in ADHD (e.g., Bollmann et al., 2017, Weigard and Huang-Pollock, 2017); we hypothesized that, for individuals with ADHD, increasing load would result in a disproportionate decrement on WM performance compared with NTs, regardless of complexity, and that this would be accompanied by increased recruitment of the fronto-striato-cerebellar networks.

Understanding the specificity of the impact of ADHD on WM capacity (complexity versus load) could elucidate what aspects of WM difficulty present a challenge for those with ADHD. Additionally, it could inform the design of personalized WM training interventions by guiding efforts toward specific aspects of WM operations. As suggested in earlier work, using external storage, cues, or incrementally adding new information may reduce WM load and interventions focused on these aspects may be more beneficial (Martinussen et al., 2005).

## 2 Material and methods

### 2.1 Participants

#### 2.1.1 Participant details

We collected imaging data (see following sections for information on imaging parameters and recruitment details) from 78 adolescents and young adults (AYA) with Combined presentation of ADHD (i.e., demonstrating elevated symptoms of both inattention and hyperactivity/impulsivity) and a comparison group of 86 NT AYA, part of a longitudinal study. We recruited participants from the University of California, Davis (UCD), MIND Institute-based subject recruitment system, UCD and community outpatient psychiatric and neurodevelopmental disorders clinics, UCD campus bulletin boards and the community via targeted advertising on flyers and social media. Twenty participants with ADHD and four NT participants were excluded due to low behavioral accuracy (defined as less than two standard deviations below the mean performance across all participants and all conditions), and 8 ADHD participants due to excessive head movement during scanning (defined as having more than 25% volumes omitted due to exceeding a volume-to-volume motion limit of 1 mm). We analyzed MRI data from the remaining participants, including 50 ADHD and 82 NT participants.

Participants were 12–23 years of age and included 41/41 and 18/32 females/males in the NT and ADHD groups, respectively (Table 1). Of the ADHD participants, 28 were currently prescribed stimulant medication (12 methylphenidate, 16 amphetamine), and two non-stimulant medication. Participants prescribed medication took a 48–96 hr medication holiday prior to the functional magnetic resonance imaging (fMRI) scans, with their prescribing physician’s approval, corresponding to five half-lives of the prescribed medication. See Supplemental Information section for information on socioeconomic status of the participants.

**Table 1:** Demographic, clinical and behavioral information.

Characteristics	NT (*n* = 82)		ADHD (*n* = 50)		T	*P*
	Mean	SD	Mean	SD		
Age (years)	17.03	3.36	16.09	2.70	−1.66	0.10
Sex (*n* Female)	41		18		−1.71	0.09
Race (*n*, %)						
More than one race	16	19.5%	12	24%		
American Indian or Alaskan Native						
Asian	7	8.5%				
Black or African American	3	3.7%				
Native Hawaiian or Pacific Islander						
White	53	64.6%	38	76%		
Other	2	2.4%				
Unknown						
Missing	1	1.2%				
Ethnicity (*n*, %)						
Hispanic or Latino	15	18.3%	10	20%		
Not Hispanic or Latino	60	73.2%	38	76%		
Unknown	1	1.2%	2	4%		
Missing	6	7.3%				
Full Scale IQ	114.72	10.86	110.22	13.43	−2.11	0.04
*Inattentive Symptoms	44.10	6.35	80.73	9.9	25.59	<0.001
*Hyperactive-Impulsive Symptoms	44.5	6.98	79.43	12.66	20.19	<0.001
**Reading Comprehension	108.38	12.95	109.60	15.72	0.39	0.69
**Word Reading	110.93	8.84	107.51	9.42	−2.20	0.03
**Problem Solving	113.22	13.50	108.51	14.87	−1.92	0.06
**Numerical Operations	115.98	15.70	107.33	15.55	−3.10	0.002
**Multiplication Fluency	106.07	15.54	100.88	15.41	1.88	0.06
**Math Composite	116.98	13.13	108.46	15.65	−3.22	0.002

* Ascertained by Conners’ Rating Scale – 3.

** Wechsler Individual Achievement Tests. Demographic variables for NT and ADHD groups are presented, followed by *t* statistic and *p* value for difference between groups. Numbers represent mean values and standard deviations (SD) except where indicated.

#### 2.1.2 Diagnostic procedures

Two licensed psychologists in our team (JBS and JFD) evaluated screening data to determine eligibility for the study based on the Diagnostic and Statistical Manual of Mental Disorders – 5th Edition (DSM 5). Parent (Conner-3 Parent Rating Scale – CPRS-3) and teacher rating scales (Conners-3 Teacher Rating Scale – CTRS-3) (Conners, 2008) were completed, whereas the adult participants had the Conners’ Adult ADHD Rating Scale (CAARS) with parent, spouse or close friend (primarily these were completed by parents) completing the Observer form of the CAARS on the participant. Childhood presence of ADHD for adult ADHD participants was also confirmed (or absence for NT) via retrospective rating scales completed by parents on the Barkley Adult ADHD Rating Scale--IV (BAARS-IV). A licensed psychologist from our team further interviewed parents to clarify diagnosis (or its absence) if needed. See below for screening procedures for academic learning disabilities.

#### 2.1.3 Study inclusion/exclusion criteria

Study inclusion criteria required participants to be between the ages of 12–25 years of age, be typically developing for the NT group or meet DSM-5 criteria for ADHD, Combined or Hyperactive/Impulsive Presentation for the ADHD group. (All participants in this study ADHD group met criteria for the Combined presentation; none for the Hyperactive/Impulsive Presentation). Study exclusion criteria included (a) Full Scale IQ score < 80 (IQ score was based on the Weschler Intelligence Scale for Children (WISC-IV; *n* = 91) or the Wechsler Adult Intelligence Scale (WAIS; *n* = 41), depending on age); (b) testing positive for a mathematical or reading learning disability (Wechsler Individual Achievement Test–Third Edition (WIAT-III) scores < 80); (c) any parent-reported history of head trauma, neurological disorder or major medical problem; (d) prescribed psychoactive medication besides ADHD medications (i.e., stimulants or atomoxetine); (e) meeting DSM criteria for any other Axis I diagnosis besides ADHD, oppositional defiant disorder, or conduct disorder; (f) a positive drug screen on the day of the imaging session for illicit drugs; (g) a positive pregnancy test (female); (h) any MRI contra-indications.

We obtained informed written parental consent and consent/child assent from all participants. The UCD Institutional Review Board approved the project.

### 2.2 Imaging

We used a Siemens 3T TIM Trio MRI scanner (Siemens Medical, Erlangen, Germany) with a 32-channel head coil for imaging. T2* functional images were acquired (voxel size = 3.4 mm × 3.4 mm × 3.4 mm, slice thickness = 3.4 mm isotropic, 36 interleaved slices, repetition time (TR) = 2.0 s, excitation time (TE) = 25 ms, flip angle = 90°, matrix 64 × 64, field of view (FOV) = 220 mm). The WM task included four runs, each run comprising of 182 volumes. Additionally, MPRAGE anatomical scan was collected (TR = 1.9 s, TE = 3.06 ms, FOV = 256 mm, matrix = 256 × 256, flip angle = 7°, slice thickness = 1 mm, 208 slices). Experimental stimuli were presented using E-Prime 2.0 (Psychology Software Tools, Inc., Sharpsburg, PA).

### 2.3 Paradigm

Participants performed a version of the Picture Order Memory Paradigm (Crone et al., 2006) using an event-related design based experimental paradigm **(**Fig. 1.1**)**. In this task, each of four runs consisted of a fixation period of 4000 ms, followed by 15 trials. Each trial started with an encoding block, consisting of four images show at 1000 ms intervals. The load was varied by replacing the fourth picture with an asterisk in the 3 load trials, which participants were instructed to ignore. This was followed by a 5000 ms instruction block, during which participants were told to recall the items in the order presented (i.e. forward; F) or in reverse order (i.e., backward; B). This was the main period of interest as this was when the objects would be either maintained (forward order) or manipulated (reverse order). After a fixation period (1000 ms), a probe block occurred, during which participants recalled the objects that had been previously presented over a 8000 ms period. An inter-trial interval of 4000 ms, 6000 ms, 8000 ms (mean 6000 ms) followed each trial. Conditions were randomly distributed within a run.

**Fig. 1:**
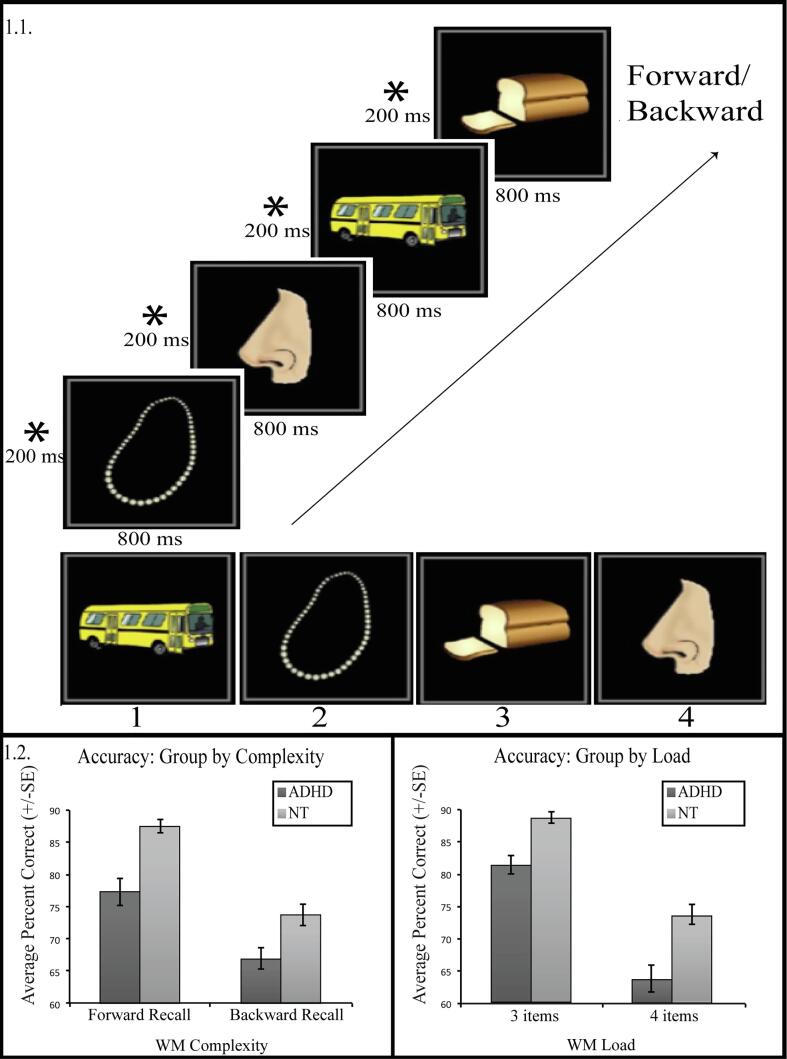
Experimental paradigm and behavioral performance. 1.1. Experimental paradigm. Each of four runs was preceded by a fixation period of 4000 ms, followed by 15 trials. Each trial started with an encoding block, consisting of four pairs of fixation, followed by an item, for 1000 ms. The load was varied by replacing the fourth picture with an asterisk in the 3 load trials, which participants were instructed to ignore. This was followed by a 5000 ms instruction block, during which participants were told to recall the items in the order presented (i.e. forward) or in reverse order (i.e. backward). This was the main period of interest as this was when the objects would be either maintained (forward order) or manipulated (reverse order). After a fixation period (1000 ms), this was followed by a probe block of 8000 ms, during which participants were asked to recall the objects that had been previously presented. An inter-trial interval of 4000 ms, 6000, 8000 ms (mean 6000 ms) followed each trial. 1.2. Behavioral Performance. The interaction between diagnosis, complexity and load was significant (*p* = 0.048). We found a significant interaction between diagnosis and load (*p* = 0.04), but not diagnosis and complexity (*p* = 0.62). Individuals with ADHD produce more errors, compared to NT, across conditions. Both groups responded less accurately for more difficult tasks – either due to increased load (4 versus 3) or increased complexity (backward versus forward, or manipulation versus maintenance), but the ADHD, versus NT group, showed greater drop in accuracy due to increased load.

### 2.4 Behavioral performance analysis

We used SAS version 9.4. (SAS Institute Inc., Cary, NC) to analyze behavioral performance. We derived average accuracy and reaction time for the 3 item (3F and 3B), 4 item (4F and 4B), forward (3F and 4F), and backward (3B and 4B) trials. Analyses were performed using mixed-effects linear models (Laird and Ware, 1982) since data were collected repeatedly for each individual across the task-conditions (complexity and load). An advantage of this approach is the ability to directly model heterogeneous variances (across groups or conditions). We tested for differences in accuracy with complexity (manipulation versus maintenance), load (4 versus 3), and diagnosis (ADHD versus NT) as factors. The model included fixed effects for diagnosis, load, complexity, age (mean-centered), the interactions between load, complexity, and diagnosis, load and diagnosis, complexity and diagnosis, load and age, complexity, and age. We also examined the quadratic effect of age. Random effects for each participant were also included.

### 2.5 Imaging analysis

#### 2.5.1 Preprocessing

We analyzed fMRI data using FSL and AFNI (Cox, 1996). The first two volumes from each scan were discarded for signal stabilization. Runs underwent non-brain removal before alignment to an individual’s T1-weighted structural MR image and transformation to Montreal Neurological Institute (MNI) space. Registration used FMRIB’s Linear Image Registration Tool (Greve and Fischl, 2009). Smoothing, using 4 mm full-width at half-maximum (FWHM) Gaussian filter, and normalization were performed as in our previous studies (Fassbender et al., 2011). Voxel size was 2 mm^3^. Volumes exceeding a volume-to-volume motion in excess of 1 mm were excluded from further analysis. Participants with more than 25% omitted volumes were excluded.

#### 2.5.2 Regression analysis

General linear model analyses fit hemodynamic responses with a boxcar activation function using onset times of each condition. Movement parameters were also included as nuisance-variables. Regressors modeled encoding, instruction, recall, and manipulation periods.

#### 2.5.3 Within and between group analysis

To identify brain regions recruited for WM complexity and load in each group, factoring out the effect of age, we carried out a linear mixed-effects modeling analysis, implemented by 3dLME in AFNI, at the whole-brain level. The fixed effects in our model were diagnosis, complexity, and load. We included interactions between diagnosis, complexity and load, diagnosis and complexity, diagnosis and load, age and load, age and complexity, age and diagnosis. Participant was treated as a random intercept. Age was included as a covariate.

We conducted Monte Carlo simulations to correct for multiple comparisons with a voxel-level *p*-value of 0.005, resulting in a minimum cluster size of 182 voxels required to achieve a probability of 0.05 of significant cluster surviving by chance. Simulations were calculated using 3dClustSim with autocorrelation function (ACF), avoiding assumptions about Gaussian noise distribution (Cox et al., 2017). Parameter estimates from significant clusters, resulting from ANCOVAs, were extracted and plotted (for demonstration only), to represent differences between groups and task-conditions, accounting for age.

To ensure that group differences were not influenced by head motion, we compared average movement parameters (calculated from square-root of sum of squares of movement in x, y, z directions) between groups, using independent samples t-tests (two-tailed, equal variances not assumed). No significant group difference was found (*t* = -0.12, df = 102.26, *p* = 0.90).

## 3 Results

### 3.1 Behavior

Table 2 and Fig. 1.2 summarize the results of the behavioral analyses testing for the effects of complexity and load on accuracy. As the table illustrates, the interaction between diagnosis, complexity and load was significant (*p* = 0.048). We found a significant interaction between diagnosis and load (*p* = 0.04), but not diagnosis and complexity (*p* = 0.62). We found a significant effect of age (*p* = 0.03). The interaction effect of age and load was significant (*p* < 0.001). We also tested for a quadratic effect of age on performance, but it was not significant (*p* = 0.06) and thus was not included as a term in fMRI data analyses.

**Table 2:** Parameter estimates from the linear mixed-effects model analysis for accuracy between groups (NT versus ADHD), complexity (manipulation versus maintenance, or backward versus forward) and load (4 versus 3), with age as a covariate. The reference categories were neurotypical for diagnosis, maintenance for complexity, and 3 items for load.

	Estimate	*SE*	*P*-value
*Behavioral Accuracy*			
*Intercept*	95.22	1.42	<0.001
*Diagnosis*	−6.55	2.05	0.002
*Complexity*	−11.41	1.23	<0.001
*Complexity X Diagnosis*	−0.98	1.99	0.62
*Load*	−12.77	1.67	<0.001
*Load X Diagnosis*	−5.56	2.72	0.04
*Complexity X Load*	−4.41	2.35	0.06
*Diagnosis X Complexity X Load*	7.60	3.82	0.048
*Age*	0.61	0.28	0.03
*Age X Age*	−0.15	0.08	0.06
*Load X Age*	1.18	0.29	<0.001

### 3.2 Brain-activation

#### 3.2.1 Task condition effects

For the neuroimaging analyses, we began by testing for the main effects of load and complexity across participants, and we identified regions previously associated with WM, including the ventrolateral and dorsolateral PFC, striatum, and cerebellum. A conjunction analysis of the main effects of load and complexity identified large parts of the occipital, parietal, middle temporal gyrus, precentral gyrus, DLPFC, cerebellum, and striatum bilaterally. Additionally, a main effect of complexity included large clusters in the medial PFC, bilateral precuneus, and cerebellum. The main effect of load further included bilateral occipital gyrus, striatum, left VLPFC, and right precentral gyrus. The main effect of diagnosis included a cluster in the cerebellum, with peak activity in the declive. The main effect of age showed large, significant clusters with peaks in the left lentiform nucleus and including the bilateral caudate, bilateral cerebellum extending over the uvula and culmen, bilateral inferior frontal gyrus (IFG), precentral gyrus, middle frontal gyrus and bilateral inferior parietal lobule (Fig. 2.1, Table 3.1).

**Fig. 2:**
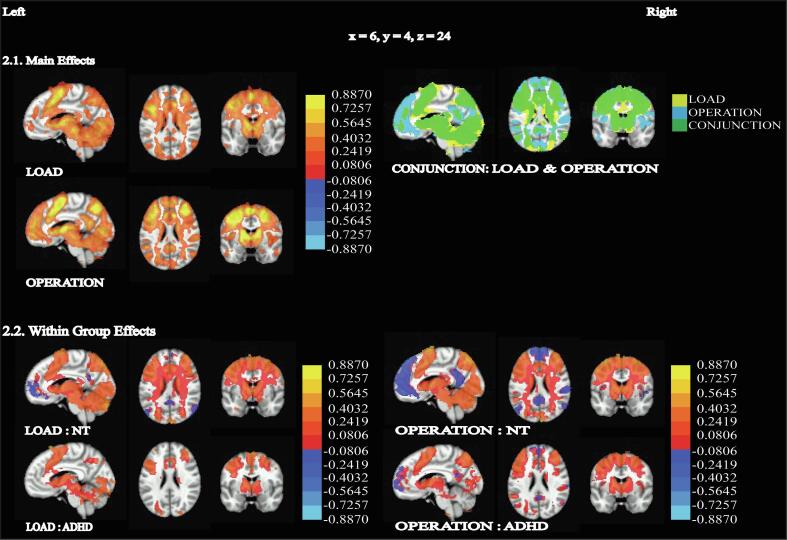
Main effects and within group effects – all images show percent signal change (equivalent to beta values) overlaid on brain images, thresholded at *p* < 0.005, cluster corrected at *p* < 0.05. All activation images except the conjunction use heatmaps to show positive activation of varying intensity from red to yellow and negative activation in shades of blue 2.1. Main Effects of load (4 *vs*. 3), complexity (backward *vs*. forward) and conjunction of the two main effects. The conjunction map shows load in yellow, operation in cyan and the overlap of the two main effects in green, 2.2. Effect of load (4 *vs*. 3) separately for NT, Effect of load (4 *vs*. 3) separately for ADHD, Effect of complexity (backward *vs*. forward) separately for NT and Effect of complexity (backward *vs*. forward) separately for ADHD. (For interpretation of the references to colour in this figure legend, the reader is referred to the web version of this article.)

**Table 3:** Comparing brain activity between groups (NT versus ADHD) for complexity (manipulation versus maintenance, or backward versus forward) and load (4 versus 3), with age as a covariate, using repeated measures ANCOVA, as implemented by 3dLME in AFNI 3.1) Main effects of group, load, complexity and age; 3.2) Load and complexity within group; 3.3) Interaction effects between group; 3.4) Interaction effects with age.

Brain Region	Brodmann Area	Hem	Volume	Peak
***3.1) Effects of Load, Complexity and Age***				

***Load (4 versus 3)***				
ITG, extends over Caudate, Cerebellum	20	L	100,607	36,42,−26
SFG, DLPFC, extends bilaterally	8,9	R	1509	−14,-52,42
Angular Gyrus	39	R	688	−54,68,30
MFG, IFG, VLPFC	10,11	R	468	−8,−68,30
Middle Temporal Gyrus	39	L	363	54,70,22
Posterior Cingulate Gyrus, Precuneus	30,23	L	361	6 56 18
SFG, MFG	6,8	L	251	10–40 44
MFG, IFG, VLPFC	11,47	R	233	−44,−36,−16

***Complexity (manipulation* versus *maintenance, or backward* versus *forward)***				
Cerebellum, Culmen		R	88,984	−30,60,−38
SFG, MFG, DLPFC	6,8,9	R	9142	−14,−34,52
Postcentral Gyrus, Angular Gyrus,IPL,Insula	13,40,42	R	2240	−54,26,20
PCG, Precuneus	30,23	L	1668	6,56,18
Insula, Precentral Gyrus, Rolandic Operculum	13	L	1420	40,4,12
Angular Gyrus, Middle Occipital Gyrus, IPL	39,40	R	829	−52,66,32
IFG,MFG,VLPFC	47,11	R	700	−40,−32,−14
Angular Gyrus, Precuneus, MTG, IPL	39	L	690	50,72,32
IFG,MFG,VLPFC	47,11	L	386	36,−26,−18
ITG, Fusiform Gyrus, MTG	20,21	L	293	52,4,−32
***Diagnosis***				
Cerebellum (with peak in the declive, extending over the tonsil, tuber, pyramis and uvula)		R	270	−32, 76, −28

***Age***				
Lentiform Nucleus, Caudate	34	L	1766	18, 2, −6
Precentral gyrus	6	L	887	28, 14, 66
Medial Frontal Gyrus	6	R	483	−2, 2, 60
Cerebellum, Uvula		L	477	30, 64, −32
Precentral Gyrus, IFG	6	L	279	54, 0, 30
Precentral Gyrus, IFG	6	R	283	−38, 8, 30
MFG	6	R	238	−36, 0, 58
Cerebellum, Culmen		R	213	−30, 58, −34
IPL	40	L	189	36, 46, 44
IPL	40	R	192	−44,34,34

***3.2) Load and Complexity within group***				

***Load (4 versus 3), NT***				
Cerebellum Declive, extends over Caudate,		R	93,629	−60,60,−34
SFG, DLPFC	9,10	R	2673	−14,−66,32
Angular Gyrus, IPL, MTG	39,19	R	581	−54,72,34
PCG	23,30	L	326	0,50,20
Angular Gyrus, IPL, MTG	39,19	L	204	52,76,24

***Load (4 versus 3), ADHD***				
SFG	6	L	42,598	6,−22,70
MFG, DLPFC	9,10	R	1108	−38,−46,32
SFG , VLPFC	11	L	587	34,−62,−14
Middle Temporal Gyrus	21,22	L	394	70,36,−6

***Complexity (manipulation versus maintenance, or backward versus forward), NT***				
Cuneus, extends over bilateral cerebellum and caudate	19,18	R	85,602	−4,98,24
SFG, DLPFC, extends bilaterally	8,9	L	8419	10,−58,44
IPL	40,1,2	R	1615	−70,28,30
PCG, extends bilaterally	23,30	L	1549	0,48,22
Insula, Claustrum	21,13	L	853	40,14,−8
Angular Gyrus, IPL	39	R	639	−54,70,36
Precuneus, IPL	19	L	529	44,76,42
IFG, VLPFC	47,11	R	418	−40,−30,−18
IFG, VLPFC	47,38	L	294	50,−28,−16

***Complexity (Manipulation versus Maintenance, or backward versus forward), ADHD***				
SFG	6	R	27,844	−16,−2,78
Cerebellum	19	R	14,924	−54,72,−28
SFG	6,8	R	4272	−14,−32,66
Posterior Cingulate Gyrus, Precuneus	31,23	R	659	−2,48,26
Cerebellum, Tonsil		R	574	−30,32,−54
STG, Insula	13,22	L	508	46,12,0
IPL, Insula	13,41	R	460	−70,28,30
Angular Gyrus, IPL	39,40	R	284	−52,68,34
Insula, STG	13,22,21	R	283	−44, 4, −6
STG, IFG, VLPFC	38,47	R	246	−46, −26, −20
Angular Gyrus, IPL	39	L	237	52, 72, 34
Cerebellum, Tonsil		L	231	16, 40, −58

***3.3) Interaction Effects Between Group***				

***Load (4 versus 3), ADHD vs. NT***				
Caudate, Striatum	45	R	523	−40, −20, 2

***Complexity (manipulation versus maintenance, or backward versus forward), ADHD vs. NT***				
Cerebellum (with peak in the culmen, extending over the tonsil, tuber, pyramis and uvula)	36	R	586	−36, 34–36
Lingual Gyrus	17	L	188	8, 96, −8

***3.4) Interaction Effects with Age***				

***Interaction between Load and Age***				
Postcentral Gyrus,Paracentral Lobule	4,5	L	409	4, 44, 68

***Interaction between Complexity and Age***				
Striatum, Caudate		R	203	−14, −18, −4

***Interaction between Group and Age***				
No significant regions found				

**Note:** DLPFC Dorsolateral Prefrontal Cortex, VLPFC Ventrolateral Prefrontal Cortex, VMPFC Ventromedial Prefrontal Cortex, MFG Middle Frontal Gyrus, IFG Inferior Frontal Gyrus, ITG Inferior Temporal Gyrus, MTG Middle temporal gyrus, STG Superior Temporal Gyrus, IPL Inferior Parietal Lobule, SFG Superior Frontal Gyrus, SPL Superior Parietal Lobule

#### 3.2.2 Within group effects

Within both groups, tests for the effect of load and complexity identified significant activation bilaterally in standard WM regions, including lateral PFC, parietal cortex, striatum, and cerebellum (Fig. 2.2, Table 3.2).

#### 3.2.3 Interactions: group × task-condition

**Fig. 3:**
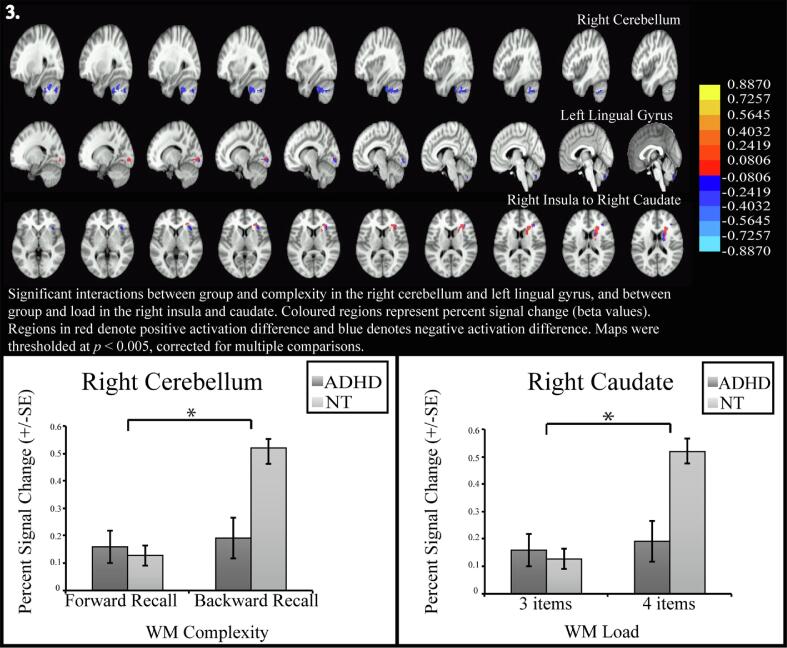
Interaction effects on brain activation between group (NT vs ADHD) and WM complexity (manipulation versus maintainence) and between group and load (3 versus 4) – all images show percent signal change (equivalent to beta values) overlaid on brain images, thresholded at *p* < 0.005 cluster corrected at *p* < 0.05. All activation images except the conjunction use heatmaps, with positive activation in red and negative activation in blue. Graphs show parameter estimates from significant clusters, extracted and plotted for demonstration purposes only. Significant interactions between group and complexity in the right cerebellum and left lingual gyrus, as well as group and load in the right insula and caudate derived using 3dLME in AFNI. We have displayed a series of adjacent slices to demonstrate the extent of the large clusters, especially the one extending from the peak in the insula across the caudate. (For interpretation of the references to colour in this figure legend, the reader is referred to the web version of this article.)

We did not find a significant three-way interaction effect (group × load × complexity). A significant interaction effect of group and complexity was found in the right cerebellum and in the left lingual gyrus. We also found a significant interaction effect of group and load in the right caudate (Fig. 3**,** Table 3.3).

#### 3.2.4 Interactions: age × task-condition

There was a significant interaction effect of age and load in the left paracentral lobule, and for age and complexity in the right caudate (Table 3.4).

#### 3.2.5 Interactions: age × group

There was no significant interaction effect of age and group.

## 4 Discussion

WM deficits have been widely reported in ADHD (Alderson et al., 2013), and they have been linked to symptoms (Rapport et al., 2009) as well as functional outcomes (Simone et al., 2018, Fried et al., 2019, Kofler et al., 2011, Orban et al., 2018, Rapport et al., 2009, Campez et al., 2020). WM impairments have also been shown to persist into adulthood (Alderson et al., 2013). Nevertheless, despite the prominence of WM-related impairments in ADHD, it is unclear whether these WM deficits are driven by increases in WM load or operational-complexity, or both. The change in neural activation accompanying an increase in WM load, versus the brain activation corresponding to greater operational-complexity are also not known, in ADHD versus NT.

Our results demonstrate that across all conditions, individuals with ADHD produce more errors, compared to NT. Both groups responded less accurately for more difficult tasks – either due to increased load (4 versus 3) or greater complexity (backward versus forward, or manipulation versus maintenance). However, in the ADHD group, increase in load had a greater impact on WM performance accuracy, compared to the NT group.

The neural data showed that all participants recruited brain regions that are typically associated with WM, such as the PFC, PC, SMA, superior temporal gyrus (D'Esposito et al., 1999), cerebellum (Tomlinson et al., 2014, Steinlin, 2007) and striatal regions (O'Reilly and Frank, 2006, Darki and Klingberg, 2015). Activity in these areas increased both with increasing load and greater complexity, suggesting significant shared neural architecture between these aspects of WM capacity. Our results suggest that maintenance at increased load, as well as manipulation, engaged the DLPFC across both groups, as in previous studies (Veltman et al., 2003, Cannon et al., 2005). We also found a significant interaction effect between operational-complexity and group in the cerebellum and in the lingual gyrus, and between load and group in the striatum. While in the simpler conditions, load or complexity, the NT group does not have significantly different activation from the ADHD group, for higher load or greater complexity, the NT group increases activation in these regions, significantly more than the ADHD group. Together the performance and brain activation differences show that those with ADHD fail to ramp up the brain activation in certain key brain regions as the task difficulty increases, but this is accompanied by a reduction in behavioral performance, compared to NT, only for increase in WM load. This suggests that load could have a greater impact than complexity on WM in ADHD. Accordingly, we also found a significant interaction between group, load and complexity for behavioral accuracy, which could reflect this difference in the effects of load and complexity between the two groups, but we did not find a corresponding interaction effect in the brain activation.

Across groups, older participants responded more accurately for all conditions, consistent with the common finding that WM improves with age (Jolles et al., 2011, Crone et al., 2006). Additionally, task accuracy dropped less in response to increasing task load for older compared to younger participants, across both groups. Several brain regions showed effects of age, including bilateral caudate, cerebellum, and some frontal regions and inferior parietal regions. We found a significant interaction between load and age in the left paracentral lobule, and between complexity and age in the right caudate. No regions showed significant interactions with group and age, indicating that the two groups are not affected differently by age in this analysis.

The lingual gyrus has been associated with encoding complex images (Machielsen et al., 2000) or words (Mechelli et al., 2000). Earlier fMRI studies of WM in ADHD have shown activation differences in the lingual gyrus. However, the direction of difference is mixed, which could be due to differences in the task employed.

Our results indicate that the caudate and the cerebellum may play an important role in WM impairments in ADHD, for load and complexity respectively. The contribution of the striatum and the cerebellum to WM has been highlighted in earlier studies (Tomlinson et al., 2014, O'Reilly and Frank, 2006, Lewis et al., 2004, Middleton and Strick, 1994, Watson et al., 2014). The striatum is hypothesized to control information-flow into WM (O'Reilly and Frank, 2006), and fMRI WM tasks have demonstrated recruitment of caudate (Lewis et al., 2004) and cerebellum (Tomlinson et al., 2014). Cerebellar damage has also been associated with WM impairments (Tomlinson et al., 2014). We further investigated the functional parcellation of the cerebellum cluster as demonstrated by (Buckner et al., 2011), where the cerebellum was parcellated based on connectivity to key brain networks, using the Yeo-7 network framework (Yeo et al., 2011). The peak of our cerebellar results was in the part most highly connected to the salience networks. However, this large cluster also extended over the limbic, visual, sensorimotor networks and fronto-parietal control network. The limbic, visual and sensorimotor networks are associated with emotional, visual and motor processing. The salience network is linked to prioritizing salient stimuli and recruits appropriate functional networks (Menon and Uddin, 2010, Bressler and Menon, 2010). The fronto-parietal control network is a control network which interacts with and manages tasks and other networks to support goals (Marek and Dosenbach, 2018).

Due to the proposed role of the striatum in gating information into WM (Chatham and Badre, 2015, McNab and Klingberg, 2008), our results showing an inability of the ADHD group to ramp up striatal activity with load may indicate a failure to scale up performance. As the cerebellum is linked to performing tasks with greater WM complexity (Marvel and Desmond, 2012), lower activation of the cerebellum for higher complexity in the ADHD group, might represent inability to scale up recruitment of this region up to match greater complexity. However, we do not see this reflected in performance, which could be driven by the greater difficulty presented by the manipulation task, especially at high load, for all participants.

The importance of fronto-striato-cerebellar networks in ADHD, across modalities, has been repeatedly highlighted (Martinussen et al., 2005, Valera et al., 2007, Hoogman et al., 2017, van Ewijk et al., 2012, Giedd et al., 2001, Casey et al., 2007, Castellanos et al., 2002). Specifically, volumetric reductions have been observed in the cerebellum (Valera et al., 2007, Baldaçara et al., 2008, Berquin et al., 1998, Wyciszkiewicz et al., 2017, Seidman et al., 2005) and caudate (Valera et al., 2007, Castellanos et al., 2002, Seidman et al., 2005, Frodl and Skokauskas, 2012); along-with lower white matter integrity in the fronto-striatal-cerebellar networks (Nagel et al., 2011) in children with ADHD, compared to NT. Functionally, WM studies of both children (Martinussen et al., 2005) and adults (Alderson et al., 2013) with ADHD feature differences in recruitment of the fronto-striato-cerebellar networks. FMRI studies found under-activation during WM tasks in the cerebellum (Mackie et al., 2007), caudate (Martinussen et al., 2005, Fassbender et al., 2011, Roman-Urrestarazu et al., 2016) or both (Massat et al., 2012) in children with ADHD, compared to NT. In adults with ADHD, we have previously demonstrated using positron emission tomography, increased regional cerebral blood flow in more distributed regions, including the cerebellum, compared to NT (Schweitzer et al., 2004). Another WM study in adult ADHD reported cerebellar under-activation, despite no reduction in WM performance (Mechelli et al., 2000). Thus, our findings in the caudate and cerebellum, are supported by previous indications of their importance in ADHD and in WM. Differences in results between studies may be due to age of the participants, performance and task difficulty.

A strength of our study lay in our inclusion criteria which resulted in relative homogeneity in the clinical symptoms in our ADHD group; All participants were required to demonstrate clinically-impairing impulsivity, in addition to other ADHD symptoms. A potential limitation of this study is the stringent criteria of excluding participants with low performance (i.e., too few correct trials), which could bias our results towards higher-performing individuals with ADHD, limiting the clinical implications. This tradeoff was required to compare brain-activation more reliably for the majority of our population. As this study is part of a longitudinal study, we also chose to use a task with a condition where the load gave room for the participants to improve in performance (i.e., 4 load) as our participants mature and all reach adulthood, when a 3 item task may result in performance with a ceiling effect. As our current data are cross-sectional, future work should also investigate how relationships between executive function and fronto-striatal-cerebellar systems in ADHD vary longitudinally with development with respect to working memory and other critical functions. We aim to investigate these questions in the future as our longitudinal dataset grows.

There was a significant difference in intellectual functioning between our groups with the ADHD group testing at a lower intellectual level than our NT group. The disorder is associated with lower cognitive ability and full scale intellectual quotient (FSIQ) often is significantly lower in ADHD than neurotypical controls (Frazier et al., 2004). This is not surprising as working memory and other processes that demand attention during the IQ test are likely to lower the IQ score and thus, controlling for it would likely over control for ADHD in the statistical model. Importantly, the group IQs for both the ADHD and NT participants were in the average to high average range and thus, we do not think that the differences in intellectual functioning likely disadvantaged the ADHD group, greatly.

WM deficits are key in ADHD (Martinussen et al., 2005, Rapport et al., 2001). WM is linked to ADHD symptoms (Rapport et al., 2009), and WM deficits persist into adulthood (Alderson et al., 2013). Nevertheless, WM is not universally impaired in ADHD (Martinussen et al., 2005, Rapport et al., 2008, Gathercole and Alloway, 2006, Vance et al., 2013, Kofler et al., 2019, Nigg, 2005), and this heterogeneity is not fully understood. Other complicating factors may include the possibility that WM impairments in ADHD may be modality-specific. It is possible that spatial WM might be more affected than verbal (Martinussen et al., 2005); however, a recent meta-analysis found verbal WM to be impacted in ADHD (Ramos et al., 2020). Other theories suggest that WM may be impacted more in individuals with inattentive symptoms (Martinussen and Tannock, 2006), yet WM deficits are also associated with hyperactive/impulsive symptoms (Kofler et al., 2019).

A notable caveat of WM studies in ADHD is the heterogeneity in findings of WM deficits in ADHD (Martinussen et al., 2005, Rapport et al., 2008, Gathercole and Alloway, 2006, Vance et al., 2013, Kofler et al., 2019, Nigg, 2005). Although the majority of previous studies in WM find deficits in ADHD (Martinussen et al., 2005, Rapport et al., 2001), some studies have failed to find any impairment (Martinussen et al., 2005, Rapport et al., 2008, Gathercole and Alloway, 2006, Vance et al., 2013, Kofler et al., 2019, Nigg, 2005). This heterogeneity is not fully understood. One reason for the disparity in results may be that WM and ADHD are both complex and heterogeneous constructs (Martinussen and Tannock, 2006, Castellanos et al., 2002, Fosco et al., 2020) and specifics of cognitive tasks could draw on impairments of varying size. For example some studies find WM impairments to be associated more with inattentive symptoms of ADHD (Martinussen and Tannock, 2006), whereas others find them linked more to hyperactive/impulsive symptoms (Kofler et al., 2019). Additionally, WM is a multi-component system, and one of the most important models of WM involve a domain general central executive component, which controls what operations will be performed, and a domain-specific storage component (phonological versus visuospatial) (Martinussen and Tannock, 2006, Castellanos et al., 2002, Fosco et al., 2020). A recent study examining the sub-components of central executive: reordering, updating and dual-processing in ADHD, found most prominent impairments in reordering, while updating and dual-processing abilities were average or superior in most individuals with ADHD (Fosco et al., 2020). Nevertheless, Fosco and colleagues also found ADHD symptom severity to be related to the central executive abilities, taken compositely, highlighting the importance of shared processes across central executive sub-components (Fosco et al., 2020). This is further complicated by WM modality. Spatial WM may be more affected than verbal WM in ADHD as suggested by a seminal review (Martinussen et al., 2005). However, a meta-analysis found verbal WM to be impacted in ADHD (Ramos et al., 2020). In the current study, we have focused on verbal WM in individuals with combined presentation diagnosis, featuring both inattentive and hyperactive symptoms, and compared the effect of WM complexity, defined as any manipulation of information held in WM as opposed to simple maintenance, versus WM load, pertaining to the amount of information as WM load. Unpacking precisely what dimensions of WM are relevant for understanding ADHD is still in its nascency, but our work fits within a grown literature aiming to delineate areas of abnormal and normal WM function.

In conclusion, although WM is impacted in ADHD, the literature is mixed regarding the nature of the relationship between ADHD and WM (Martinussen et al., 2005, Rapport et al., 2008). That is, it was not known if all WM operations are impacted at higher loads, or if only more complex operations, such as manipulation, are affected. Most prior studies of WM in ADHD, and brain imaging studies in particular, have focused on maintenance (Martinussen et al., 2005, Roman-Urrestarazu et al., 2016, Massat et al., 2012), and none have directly compared maintenance and manipulation and different loads within the same experiment. We found that in AYA with ADHD not only more complex operations such as manipulation, but also maintenance at higher loads is impacted in ADHD. Indeed, we show that behaviorally the impact of greater load is more than increased complexity, in ADHD, although both show an impact neurally, with the ADHD group under-activating the cerebellum for greater complexity and caudate for higher load. These findings enhance the specificity of our understanding of WM deficits in ADHD by elucidating which aspects of WM difficulty are more challenging for those with ADHD. This in turn could inform the design of remedial interventions.

## Funding

This work was supported by 10.13039/100000025National Institute of Mental Health grants R01 MH091068 (Schweitzer) and U54 HD079125 (Abbeduto).

## Financial disclosures

Dr. Hinshaw receives book royalties from Oxford University Press and St. Martin’s Press. Mr. Hartanto and Drs. Mukherjee, Fassbender, Iosif, van den Bos, Guyer, Pakyurek, McClure, and Schweitzer report no competing interests.

## CRediT authorship contribution statement

**Prerona Mukherjee:** Conceptualization, Methodology, Software, Formal analysis, Data curation, Investigation, Writing - original draft, Writing - review & editing, Visualization, Project administration. **Tadeus Hartanto:** Investigation, Software, Data curation. **Ana-Maria Iosif:** Formal analysis, Writing - review & editing. **J. Faye Dixon:** Investigation, Writing - review & editing. **Stephen P. Hinshaw:** Writing - review & editing. **Murat Pakyurek:** Investigation. **Wouter van den Bos:** Writing - review & editing **Amanda E. Guyer:** Writing - review & editing. **Samuel McClure:** Conceptualization, Methodology, Writing - review & editing, Supervision. **Julie B. Schweitzer:** Conceptualization, Investigation, Writing - review & editing, Supervision, Funding acquisition, Project administration. **Catherine Fassbender:** Conceptualization, Methodology, Investigation, Writing - review & editing, Visualization, Supervision, Project administration.
