# Supplementary material for: Neural basis of working memory in ADHD: Load versus complexity
Source: Neuroimage Clin. 2021 Apr 3;30:102662. doi: 10.1016/j.nicl.2021.102662 (PMC8175567; doi:10.1016/j.nicl.2021.102662)
Supplement: Supplementary data 1 [file mmc1.docx]

### Supplemental Information

### Socioeconomic Status

### Socioeconomic status (SES) was determined based on educational attainment and income. Adult participants reported on their income and educational level. Most adults diagnosed with ADHD reported some college (72.7%) and 18.2% reported having completed high school. Of the adults included in the NT group, 82.8% reported having graduated high school or some college, whereas 13.8% reported an Associate’s or Bachelor’s degree. For income, 81.8% of adults in the ADHD group reported earning up to $25,000 and 9.1% reported an annual income of up to $50,000. Similarly, 89.7% of adults in the NT group reported an annual income of up to $25,000 and 6.9% reported earning up to $50,000. For minors, SES was based on parental report of their own educational attainment and income as well as of their partners, if applicable. Approximately 51% of parents of children in the NT group reported an Associate’s or Bachelor’s degree; 26.4% reported a Master’s or Doctoral degree, 5.7% reported a professional degree, and approximately 13.2 % reported a high school diploma or some college. For the ADHD group, 56% of parents reported an Associate’s or Bachelor’s degree; 20.5% reported a Master’s or Doctoral degree; and 20.5% reported some college or a professional degree. Household income was estimated by summing the lower and upper ends of categorical values provided by parents regarding theirs and their partner income, if applicable. Most parents in both the ADHD and NT groups reported a household income of over $100, 000 (ADHD= 65.8%; NT= 45.1%); 15.4 % of the ADHD and 35.6% of the NT group fell within the $50,000-$125,000 range, and 12.8% of the ADHD and 15.7% of the NT groups reported earning between $0-$75,000.
